# In response to: multimodal neuromonitoring in traumatic brain injury patients: the search for the holy grail

**DOI:** 10.1186/s13054-023-04728-8

**Published:** 2023-11-20

**Authors:** Teodor Svedung Wettervik, Erta Beqiri, Peter Smielewski

**Affiliations:** 1 Section of Neurosurgery, Department of Medical Sciences, Uppsala University, 751 85 Uppsala, Sweden; 2 Brain Physics Laboratory, Division of Neurosurgery, Department of Clinical Neurosciences, University of Cambridge, Cambridge, UK

We thank Taccone et al. for their important comments regarding our original article [1]. In this part I study, we explored the relation between global cerebral physiological variables (intracranial pressure [ICP], cerebral perfusion pressure [CPP], pressure reactivity index [PRx], and optimal CPP [CPPopt]) and brain tissue oxygenation (pbtO_2_) in traumatic brain injury (TBI).

We found that although the global cerebral physiological variables and pbtO_2_ were significantly related, these associations were weak in magnitude and were most pronounced in cases of extreme disturbances in global cerebral physiology. Notably, these findings were also evident in linear mixed effect models, in which we included patients as a random effect. This approach allowed us to account for patient heterogeneity in terms of differences in the proximity between focal lesions and the pbtO_2_ probe and slight changes in neurocritical care management over the long study period. The latter included approaches informed by both pbtO_2_ and PRx/CPPopt in the later years. Unfortunately, access to specific data on pbtO_2_ probe location and more granular details of pbtO_2_-directed management were not available for analysis. Thus, while we fully agree with Taccone et al. that such data would have refined our analyses, we feel that our statistical approach has lessened these effects somewhat by focusing on the within-patient relationships.

Furthermore, although ICP, CPP, PRx, and CPPopt are typically considered the main surrogates of global cerebral perfusion in the neurocritical care setting, other variables such as systemic oxygenation and cerebral energy metabolism are important modulators of brain tissue oxygenation. As indicated by Taccone et al., further studies are warranted that also include pulse oximetry, arterial blood gases, and microdialysis data to in greater detail investigate the complex pathophysiological interplay among systemic and cerebral physiological variables.

In addition, Taccone et al. raises an intriguing question if optimal cerebral haemodynamic targets should be oriented towards preservation of cerebral autoregulation or focus on down-stream variables such as achieving adequate brain tissue oxygenation. Autoregulatory-oriented therapy aims at targeting the CPP for which the brain is best protected from blood flow variations and in practical terms this implies avoidance of both ischaemia and hyperaemia. However, in special circumstances, it is possible that ischaemia and hypoxia still can take place although the CPP lies within the autoregulatory range, such as in case of severe hyperventilation or in failure of the diffusive oxygen transport in brain tissue. Thus, the advantage of pbtO_2_ or microdialysis of energy metabolism is that these tools monitor downstream physiology to CPP/CPPopt and thereby could better indicate when cerebral decompensation occurs. Still, the issue with pbtO_2_ and microdialysis is their spatial constraints and limited validity to reflect the physiology of the entire brain [2]. As outlined in the part II study [3], in which we explored the prognostic role of low pbtO_2_, hypoxic pbtO_2_ was primarily associated with outcome when it occurred in association with disturbances in other global cerebral physiological variables, but rarely in the setting of low pbtO_2_ alone. Thus, pbtO_2_ monitoring appears to be an important diagnostic adjunct to ICP, PRx, CPP, and CPPopt as an indicator of detrimental ischaemic hypoxia, while the clinical significance of isolated pbtO_2_ insults is less clear. Finally, low pbto_2_ was associated with unfavourable outcome in a multiple logistic regression in the part II study [3]. However, this association was attenuated to non-significant levels when ΔCPPopt < −5 mmHg (which turned out significant) was included in the model. Consequently, we speculate if the global autoregulatory target CPPopt may be a stronger indicator of clinically significant global cerebral blood flow disturbances than hypoxic pbtO_2_-values, due to the focal limitations of the latter monitoring tool.

Altogether, our main purpose of our part I study [1] was to determine whether pbtO_2_ is a suitable surrogate measure of cerebral haemodynamic optimization for future clinical trials on autoregulatory therapy [4]. However, most likely due to the complexity in the interaction of cerebral physiological variables and differences in monitoring techniques (global vs. focal), pbtO_2_ did not appear to be a good surrogate measure for these purposes. Still, we do indeed think that comprehensive monitoring, including pbtO_2_, and the integration of the information derived from each monitoring modality remains of outmost importance in order to describe the pathophysiology in TBI patients. However, as always in this field, we anticipate that it will be challenging to prove whether it actually benefits functional outcomes [5].

## Data Availability

Not applicable.
